# Seroprevalence of varicella-zoster virus among pregnant women in Fayoum Governorate, Egypt

**DOI:** 10.1186/s42506-018-0002-5

**Published:** 2019-01-07

**Authors:** Enas G. Ibrahim, Wafaa Y. Abdel Wahed, Hanaa M. Eid, Wessam S. Deeb

**Affiliations:** 1 Microbiology Department, Faculty of Medicine, Fayoum University, Faiyum, Egypt; 2 Public Health & Community Medicine Department, Faculty of Medicine, Fayoum University, Faiyum, 63514 Egypt; 3 Obstetric and Gynecological Department, Faculty of Medicine, Fayoum University, Faiyum, Egypt

**Keywords:** Varicella, Seroprevalence, Pregnant women

## Abstract

**Background:**

Chickenpox infection acquired during pregnancy is a serious condition. There may be congenital malformations and neonatal varicella syndrome with significant morbidity and mortality. Egypt has no routine varicella-zoster vaccination program.

**Objective:**

To assess the immune status against varicella-zoster virus (VZV) antibodies among a group of pregnant women and to study the relationship between VZV seroprevalence and some sociodemographic characteristics.

**Subjects and methods:**

A descriptive cross-sectional study was conducted on a group of pregnant women (*n* = 333) attending antenatal care (ANC) clinic at Fayoum University Hospital. Serologic testing for VZV was performed using ELISA through the years 2016–2017.

**Results:**

VZV seroprevalence was detected in 294 (88.3%) of the 333 recruited pregnant women. Older age > 25 years old was significantly associated with low percent of VZV-negative antibodies (6.7% in versus 17.4% in younger age, OR (95%CI) 0.34 (0.17–0.70)), also having more than one child was significantly associated with a low percent of VZV-negative antibodies (8.2% versus 16.1% among participants with no children or having one child, OR 0.34 (0.17–0.70)).

**Conclusions:**

Despite the absence of a routine VZV vaccination program in Egypt, VZV immunity was high among pregnant women, but less than that reported in many developed countries. We recommend targeted vaccination for women in the reproductive age especially young and primipara.

**Trial registration:**

Ethical Committee Registration number R67 session 42: date 12/11/2017(retrospectively registered).

## Introduction

Varicella-zoster virus (VZV) is an alpha-herpes virus neurotropic type. Its primary infection occurs in childhood, causes varicella (chickenpox), and then the virus becomes latent in dorsal root ganglia, cranial nerve ganglia, and autonomic ganglia along the entire neuraxis [1]. When VZV cell-mediated immunity decreases with advancing age and immunosuppression, the virus reactivates to produce herpes zoster (shingles), which is complicated by post-herpetic neuralgia (PHN, a dermatomal pain that persists after the disappearance of rash) [2].

When VZV infection occurs during pregnancy, it leads to serious outcomes; this occurs irrespective of the term of pregnancy. Congenital varicella syndrome (CVS) occurs if pregnant women are infected before 18 weeks of gestation which leads to congenital defects [3]. If pregnant women had VZV infection during the third trimester or close to delivery, this may cause infection in newborn associated with increased mortality rate of infants up to 20%. If contact occurs before and after delivery by days, the risk of chickenpox is about 17–30% if developed in neonates [4, 5].

Epidemiology of VZV varies between temperate and tropical climates. Also, varicella incidence varies with respect to population density and risk of exposure, social factors, humid conditions, and especially geographic locations of the world [6]. Talukder et al. through their research to detect VZV seroprevalence have shown that VZV antibody prevalence was 93.1% in White pregnant British women, while it was 86.8% in Bangladeshi women [7]. In Hong Kong 95.4% [8], in Iran VZV level of immunity has been reported to be 76.5 to 86.9% among females in childbearing age [6]. The difference in VZV seroprevalence between countries implies that there is a risk of varicella infection in pregnant women living in tropical areas.

VZV vaccination is not part of routine nationwide immunization of Egypt, and there is a scarcity of published studies concerning the level of protective antibody in pregnant women to VZV infection in Egypt. The aim of this study is to investigate the antibody status against one of the vaccine-preventable diseases VZV among a group of pregnant women at the Obstetric Department, Fayoum University, and to study the relationship between VZV seroprevalence and some sociodemographic characteristics of women.

## Materials and methods

### Study design and setting

This cross-sectional study was performed from September 2016 to March 2017 on pregnant women attending the antenatal care (ANC) clinic at Fayoum University Hospital (FUH), Fayoum Governorate, Egypt.

Fayoum is a large depression or basin in the southwest of Cairo, and Fayoum Governorate’s population amounts to 3.17 million persons [9]. Most of them live in rural communities and work in agriculture and its related industries. The average family size ranges from 4.1 to 4.5 from urban to rural communities respectively [10].

### Study participants

Inclusion criterion for this study was pregnant women attending the ANC outpatient clinic at FUH. Exclusion criteria were having acute varicella infection and undergoing immunosuppressive therapy. Three hundred thirty-three participants were recruited to participate in the study. The sample size was calculated, using a single proportion formula with maximum allowable error set at 5%, a proportion of positive seroprevalence of VZV antibodies at 76.5% (6), and at 5% significance level. The required number was 277. To adjust for 10% nonresponse rate, the estimated sample was increased to 305; through this research, 333 participants were recruited to participate in the study.

### Study tools

A predesigned interview questionnaire was developed to collect data concerned with demographic characteristics of participants including their age, education, occupation, number of children, and residency (rural or urban). The history of varicella infection and contact exposure were documented. Also, their awareness about the adverse effect of varicella during pregnancy was assessed.

### Laboratory and virologic examination

For the consenting subjects, an extra 5 ml of clotted blood was collected for the determination of varicella immunity status (VZIgG). After blood collection, all samples were sent to laboratory of Fayoum University and centrifuged, and serum was collected and frozen at − 80 °C until use; all CRFs were recorded into a database by an independent reviewer.

The antibody index was determined using a commercially available Varicella-Zoster IgG ELISA kit [11]. Optical density measurements were performed, and results were calculated using an automated system. All tests were evaluated using adequate positive and negative reference controls according to the manufacturer’s instructions. Results were classified as positive, negative, or equivocal. Specimens with equivocal results were retested using the same test kit, and those few that were equivocal again were classified as negative [12].

### Statistical analysis

Statistical analysis was performed using SPSS (Statistical Package for Social Sciences) version 16 (SPSS Inc., Chicago). Variables were presented using number and percent for qualitative variables, mean, and standard deviation for quantitative variables. Comparison between varicella-positive and varicella-negative patients was done using chi-square test for qualitative variables and Student’s *t* test for quantitative variables. Forward stepwise logistic regression analysis was performed to assess predictors of varicella seroprevalence. *p* value equal and less than 0.05 was considered statistically significant.

## Results

Three hundred thirty-three pregnant women were enrolled with mean age of 26.5 ± 5.6 years (ranged from 15 to 43). Around 60% of participants were from rural areas. More than two thirds of the studied sample (68.5%) had formal education at or beyond secondary school, and the majority of them were not working (93.7%). Among husbands, 71.5% of them were educated at or beyond secondary school, and 80.8% had no regular employment. More than half of participants (55.3%) had more than one child (Table 1).

**Table 1:** Sociodemographic characteristics of varicella seronegative pregnant women in comparison with seropositive, Fayoum, Egypt, 2016–2017

Variables	Total, *n* = 333	Varicella negatives, *n* = 39 (11.7)	Varicella positives, *n* = 294 (88.3%)	OR (95%CI)	*p* value
Maternal age				0.34 (0.17–0.70)	
< 25 years	155 (46.5)	27 (17.4)	128 (82.6)		0.003*
≥ 25 years	178 (53.5)	12 (6.7)	166 (93.3)		
Residence					0.234
Urban	133 (39.9)	19 (4.3)	114 (85.7)	0.67 (0.34–1.30)	
Rural	200 (60.1)	20 (10.0)	180 (90.0)		
Mother education					0.913
Less than secondary education	105 (31.5)	12 (11.4)	93 (88.6)	1.04 (0.51–2.15)	
Secondary and higher education	228 (68.5)	27 (11.8)	201 (88.2)		
Husband education				1.63 (0.72–3.7)	0.238
Less than secondary education	95 (28.5)	8 (8.4)	87 (91.6)		
Secondary and higher education	238 (71.5)	31 (13.0)	207 (87.0)		
Mother occupation				0.36 (0.05–2.76)	0.49
Not working	312 (93.7)	38 (12.2)	274 (87.7)		
Employed	21 (6.3)	1 (4.8)	20 (95.2)		
Husband occupation	269			1.3 (0.59–2.9)	0.52
Irregular work	(80.8)	30 (11.2)	239 (88.8)		
Regular work	64 (19.2)	9 (14.1)	55 (85.9)		
No of children					0.025*
One child or no children	149 (44.7)	24 (16.1)	125 (83.9)	0.46 (0.23–0.92)	
More than one child	184 (55.3)	15 (8.2)	169 (91.8)		

*Significant at *p* < 0.05

Our results showed that VZV antibody prevalence was detected among 294 (88.3) while 39 (11.7) were serologically negative for varicella antibodies. The mean age of participants with positive as compared to negative serology was 26.77 ± 5.72 versus 24.44 ± 4.13 years, respectively (*p* < 0.05). Older age (≥ 25 years old) was significantly associated with low percent of VZV-negative seroprevalence (6.7% in comparison with younger age 17.4%, OR (95%CI) 0.34 (0.17–0.70); the percent of seroprevalence of VZV antibodies was significantly higher in pregnant women with more than one child (91.8%) in comparison with those having no children or only one child (*p* = 0. 025). (Table 1).

No significant relation between varicella seroprevalence and reported medical history of diabetes, hypertension, and HCV was revealed among pregnant women (Table 2).

**Table 2:** Comparison of medical history between varicella negative and positive pregnant women Fayoum, Egypt, 2016–2017

Medical hisory	Varicella seronegatives (*n* = 39)	Varicella seropositives (*n* = 294)	*p* value
Hypertension			
Yes (12)	1 (7.7)	12 (92.3)	0.999
No (320)	38 (11.9)	282 (88.1)	
Diabetes			0.394
Yes (4)	1 (25.0)	3 (75.0)	
No (329)	31 (12.4)	211 (87.6)	
Diagnosed HCV			0.394
Yes (4)	1 (25.0)	3 (75.0)	
No (329)	38 (11.6)	291 (88.4)	

No significant relation between varicella seroprevalence and any reported history of varicella infection or contact with varicella patient was detected (*p* > 0.01) (Table 3).

**Table 3:** Comparison of history of varicella exposure among pregnant women in relation to varicella seroprevalence

Varicella exposure	Varicella seronegatives (*n* = 39)	Varicella seropositives (*n* = 294)	*p* value
History of varicella infection			
Yes (48)	5 (10.4)	43 (89.6)	0.78
No (241)	30 (12.4)	211 (87.6)	
Do not know (44)	4 (9.1)	40 (90.9)	
History of varicella contact			0.79
Yes (40)	5 (12.5)	35 (87.5)	
No (293)	34 (11.6)	259 (88.4)	

By multivariate analysis forward stepwise method, the only predictor for negative seroprevalence was women aged less than 25 years old versus older age (≥ 25 years old) (Table 4).

**Table 4:** Predictors of negative seroprevalence of varicella among study women (forward stepwise logistic regression)

Predictors	*p* value	OR (95%CI)
Age group < 25 years versus ≥ 25 years old	0.003	1.7 (1.19–2.4)

**Fig. 1:**
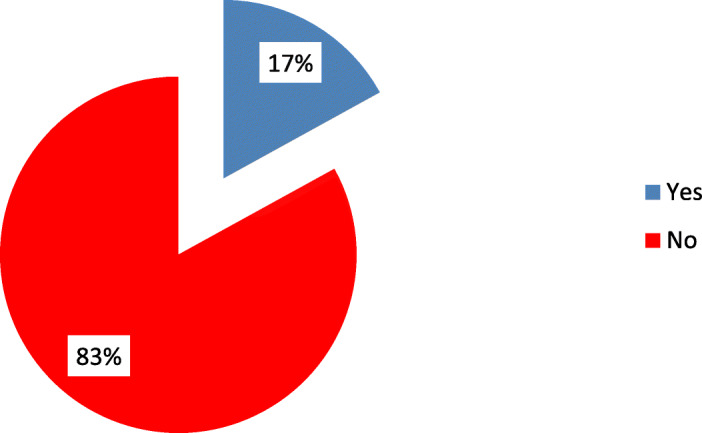
Awareness of pregnant women of adverse effects of varicella on pregnancy, Fayoum, Egypt, 2016–2017

The majority of our participants were unaware of the effect of varicella infection on the outcome of pregnancy (Fig. 1).

## Discussion

Varicella infection during pregnancy may result in VZV transmission to the fetus or newborn with serious complications such as spontaneous abortion, premature labor, varicella pneumonitis, premature delivery, and CVS [3].

In the current study, varicella seroprevalence was detected among 88.3% of pregnant women. This was consistent with studies with high VZV seroprevalence reported in South Korea (92.7%), Israel (90.2%), Italy (80.9%), Iran (90.3%), and Lyon, France (98.8%) [13–17] while it is inconsistent with low VZV seroprevalence reported in India (68.2%) [18], Singapore (55.3%), [19], and Pakistan (41.8%) [20].

The differences between countries are likely attributed to climatic conditions and introduction of varicella vaccine in developed countries. The country of birth and country in which childhood was spent are more important in determining chickenpox immunity, as the seroprevalence of UK-born Bangladesh women (95.2%) was similar to that in UK-born Caucasians (93.1%), and which was much higher than Bangladesh women born and grew up in Bangladesh (84.6%) [5, 7].

Although Egypt is a tropical climate developing country and no routine VZV vaccination program was implemented, VZV seroprevalence was high (88.3%). This may be due to the older age group of our participants ranged from 15 to 43 years old who became immune due to previous VZV infection or contact in our overpopulated country. A recent study conducted on Egyptian primary school children has reported VZV seroprevalence to be 38.9% and correlated increasing VZV antibodies with increasing age [21].

Our results demonstrated a close relationship between seroprevalence and age. Seroprevalence was positive in 82% of pregnant females less than 25 years versus 93.3% of pregnant females more than 25 years. This is probably related to the dense population and close contact among children in nurseries and schools that would promote its transmission in Egypt. Similar age-related results were systematically reported in North America-, Europe-, and Asia-Pacific-based studies [22]. Age-dependent VZV seropositivity was also demonstrated in European population [23].

Our results demonstrated no difference in seroprevalence between rural and urban areas. This was consistent with previous studies [6, 24] and inconsistent with other studies that reported rural/urban difference [25, 26].

The present study assessed the role of self-recalled history of chickenpox in determining varicella seroprevalence. Our results reported no significant relation between varicella seroprevalence and any reported history of varicella infection or contact varicella patient. Thus, self-recalled history is highly not a reliable method in considering serologic evidence of varicella immunity. This was in opposition to what was reported in Iran that self-reported positive history of varicella to be a strong predictor of varicella immunity [1].

Awareness of chickenpox adverse effect on pregnancy was limited in our obstetric population. Our results suggested that 83% of pregnant women were unaware of the adverse effect of varicella on pregnancy, and only 17% are aware of an adverse effect which draws the attention to the importance of educating and increasing females’ awareness.

## Strengths and limitations

Our results may represent a true background evaluation of the immune status of females in childbearing years to VZV infection in Egypt, who are at risk of developing severe varicella and its complication. Although the majority of pregnant women are immune, still 12% are susceptible to VZV infection. Vaccination of susceptible women in childbearing years is recommended and advocated to avoid the risk of perinatal and congenital varicella in addition to the chance of nosocomial exposure of health care worker to infection [27]. A screening program for all women in reproductive age may be costly and inappropriate, so we can recommend vaccinating women having one or all of the following criteria: (i) less than 25 years, (ii) nulli- or primipara, and (iii) have no history of varicella infection.

Limited memory and understanding of our pregnant population may be the reason behind unreliable data related to the reported history of varicella infection or exposure. Also, because of the lack of accurate recall, we could not represent our results related to the age of varicella infection or status of receiving varicella vaccine.

## Conclusions and recommendations

Despite the absence of a routine vaccination program in Egypt, VZV immunity was high among pregnant women but less than reported in many developed countries that introduced VZV vaccination. We cannot depend on the self-reported history of chickenpox in determining varicella susceptibility. We recommend targeted vaccination for women in the reproductive age especially young and primiparous women.
